# Dr. Broussais

**Published:** 1839-01

**Authors:** 


### OBITUARY.

### BROUSSAIS.

Fr. Jos. Vict. Broussais was born at St. Malo, on the 17th December, 1772. Being the son of a medical man he received some professional instruction under his father's roof; but, upon the whole, his education, as well medical as of a more general kind, was very imperfect; and when he entered the public service, as an assistant surgeon, in his twentieth year, there is little doubt that he had yet to learn the art which he was about to practise. He first joined the marine service, and served in it ten years: he then proceeded to Paris to prosecute his studies, and took his degree of M.D. in the year 1803. He remained in Paris two years longer, and then joined the army, with which he served until the peace of 1814, being in the campaigns of Germany, Holland, Italy, and Spain. On his return to Paris, he was appointed physician-in-chief and professor of the military hospital of Val-de-Grace, to which he remained attached many years, promulgating his peculiar doctrines, with the utmost energy, to a large body of pupils. In 1831, he was appointed professor of general pathology in the Academy of Medicine. He was also a commander of the Legion of Honour. He died at his country-house at Vitry, on the 18th November, 1838, rather suddenly at last, although he had long laboured under cancer of the rectum. He was buried with great pomp at the Père-la-Chaise on the 21st November, the students of medicine themselves dragging the hearse in honour of the deceased.

Another opportunity will probably be afforded to us of speaking of the professional character of M. Broussais, and of the doctrines which will ever render his name memorable in the history of medicine. These doctrines, regarded as a system of medical philosophy, are fast falling to the level to which they belong; but like almost all other systems, that of Broussais has left behind it not a little good. More than any other founder of a medical sect in modern times, Broussais has influenced medical practice in every civilized country; and it can hardly be doubted, that, although many of his doctrines were false and much of his practice bad, the general influence of his system has been beneficial, in a practical point of view.

M. Broussais' publications are numerous and well known. His greatest work is *The History of Chronic Inflammations*, which will retain its place as a valuable treatise in practical medicine, when all his theories are merely recognized as matters of history.

The following is as complete a list of M. Broussais' writings as we have been able to procure: we shall arrange them in chronological order.

1805. Thèse sur la Fièvre hectique.
1808. Histoire des Phlegmasies chroniques. 2 vols.
1811. Lettre sur le Service de Santé interieur.
1816. Examen de la Doctrine médicale généralement adoptée.
1821. Examen des Systèmes de Nosologie, précedé de Propositions renfermant la Substance de la Médecine physiologique.
1824. Traité de Physiologie appliquée à la Pathologie.
1824. Catéchisme de la Médecine physiologique.
1826. De la Théorie médicale, dite pathologique.
1828. De l'Irritation et de la Folie.*
1829. Commentaires des Propositions de Pathologie consignées dans l'Examen. 2 vols.

* A second edition of this work, in two volumes, was passing through the press at the time of the author's death.

1829. Réponse aux Critiques de l'ouvrage sur l'Irritation et la Folie.
1832. Mémoires sur la Philosophie de la Médecine et sur l'Influence des Médecins physiologiques.
1832. De Cholera-Morbus épidemique.
1834. Mémoire sur l'Association du Physique et du Moral, (Mémoires de l'Académie, vol. I.)
1835. Cours de Pathologie et de Thérapeutiques générales.* 5 vols.
1836. Cours de Phrénologie. 1 vol.

Besides the above, there are many articles from the pen of Broussais in the "Annales de la Médecine physiologique," a journal established by him (1822-1834); and a few in the "Journal de Phrénologie."

